# Development of muffins as dialysis snacks for patients undergoing hemodialysis: results of chemical composition and sensory analysis

**DOI:** 10.1007/s40620-020-00831-z

**Published:** 2020-08-28

**Authors:** Jessica Machado, Roberta Fontanive Miyahira, Monica Marques, Nathalia Moura-Nunes, Renata Rangel Guimarães, Lilia Zago, Isabelle Santana, Maurilo Leite Junior, Carla Maria Avesani

**Affiliations:** 1 Graduation Program in Food, Nutrition and Health, Institute of Nutrition, Rio de Janeiro State University, R São Francisco Xavier, 524, Rio de Janeiro, RJ 20550-900 Brazil; 2 Department of Basic and Experimental Nutrition, Institute of Nutrition, Rio de Janeiro State University, R Sao Francisco Xavier, 524, Rio de Janeiro, 20550-900 Brazil; 3 Department of Organic Chemistry, Institute of Chemistry, Rio de Janeiro State University, R Sao Francisco Xavier, 524, Rio de Janeiro, 20550-90 Brazil; 4 Department of Clinical Science, Intervention and Technology, Division of Renal Medicine, Baxter Novum, Karolinska Institute, M99 Karolinska University Hospital Huddinge, Huddinge, 14186 Sweden; 5 Nephrology Division, Rio de Janeiro Federal University, Av Pedro Calmon, 550, Rio de Janeiro, 21941-901 Brazil

**Keywords:** Hemodialysis, Dialysis snack, Protein energy wasting, Energy protein supplement

## Abstract

**Objective:**

This study aimed to develop two non-industrial food products as financially accessible options to prevent and treat malnutrition in hemodialysis (HD) patients. These food products were developed and intended for use as dialysis snacks.

**Methods:**

This is a cross-sectional and multi-step study. First, 183 adult HD patients (55 ± 14 years; 50.8% males), replied to a questionnaire with their food preferences regarding taste (salty, sweet, bitter, sour) and consistency (liquid, solid, pasty) for a dialysis snack. Most patients preferred a food product with a solid consistency (90%) and a salty flavor (81.4%). Second, three muffin formulations of fine herbs were developed; one enriched with whey protein concentrate (WPC), a second with textured soy protein (TSP) and a third standard formulation without protein for comparison with the protein-enriched muffins, for which the chemical and nutritional compositions were analyzed. In the third step, 60 patients on HD (61 ± 15 years; 53% males) were enrolled in a sensory analysis by applying a 9-point structured hedonic scale, ranging from “extremely liked” (score 9) to “extremely disliked” (score 1).

**Results:**

When compared with the standard formulation, the formulations enriched with WPC and TSP protein had a significantly higher amount of protein/serving (Standard: 5.9 ± 0.3 g vs WPC: 14.5 ± 0.9 g and TSP 10.8 ± 0.7 g; P < 0.05) but a lower amount of carbohydrate (Standard: 13.1 ± 2.2 g vs WPC: 5.6 ± 0.8 g and TSP 6.0 ± 1.2 g vs; P < 0.05). The mineral content/serving of the protein-enriched muffins was low in phosphorus (50 mg) and sodium (180 mg). The potassium content/serving was moderate for the WPC muffin (225.2 mg) and low for the TSP muffin (107.9 mg). The acceptability index (AI) for the enriched protein muffins was higher than 70% and similar to the standard formulation.

**Conclusion:**

The muffins with fine herbs and enriched with protein were well-accepted by all patients and appropriate to serve as dialysis snacks for HD patients.

## Introduction

Malnutrition is a prevalent condition in patients with chronic kidney disease (CKD) undergoing dialysis. According to a recent meta-analysis including studies from several countries, in which malnutrition has been diagnosed either by the 7-point subjective global assessment (SGA-7p) or the malnutrition inflammation score (MIS), 28–54% of the dialysis patients had malnutrition [1]. The etiology of malnutrition is multifactorial, including disease and treatment-related factors that, together, create a condition where reduced energy and protein intake are concomitantly observed, with increased protein catabolism and energy expenditure [2]. This particular condition led the expert panel of the International Society in Renal Nutrition and Metabolism (ISRNM) to use the term protein energy wasting (PEW) to describe the nutritional disorder that occurs in CKD [3]. Importantly, Ikizler et al. [4] have shown that energy expenditure and protein degradation increase substantially during and after a 2-h hemodialysis (HD) session, leading to a negative energy and protein balance. In a subsequent study, the same group showed that intradialytic oral energy-protein supplementation was capable of reversing the acute energy and protein catabolism effect caused by the dialysis procedure [5]. These findings reinforce the importance of providing energy and protein support to attenuate the protein catabolism that occurs during and after HD.

Several studies have shown the effectiveness of industrial oral nutrition supplements [6, 7] in order to compensate for the reduced food intake, reported mainly on HD days [8–10]. In addition, a potential benefit of oral supplementation to increase survival [6, 11] and to decrease missed HD treatments [6] has also been documented. Nevertheless, the use of industrial intradialytic oral supplementation is not feasible for many dialysis centers due to its high cost for long-term use. Moreover, low adherence to such supplements has been reported, likely due to the monotony of taste and flavor [12]. In this sense, non-industrial supplements that can be prepared by the patient, family or caregivers are likely to confer advantages for long-term use, in addition to allowing for more independence in the treatment and greater dietary flexibility.

The present study set out to develop two food products with high energy and protein density, whilst remaining moderate in potassium (K), phosphorus (P) and sodium (Na), and that could be made using reasonably-priced ingredients. Such products could be feasible and affordable options for use as dialysis snacks to both prevent and treat PEW in HD patients.

## Methods

### Study design and protocol

**Fig. 1:**
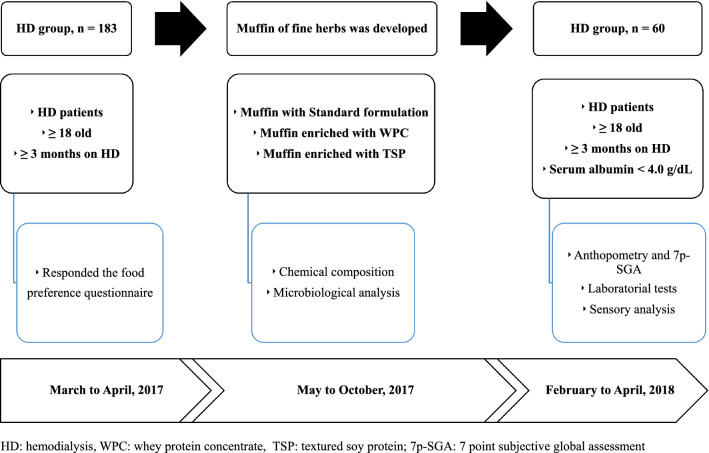
Study protocol describing patient selection, development of the muffins and sensory analysis

This is a cross-sectional study including two samples of adult patients (aged ≥ 18 years) undergoing chronic HD treatment, on a regular 3 sessions-per-week schedule for at least 3 months, from two dialysis centers located in Rio de Janeiro, Brazil. The first sample was made up of 183 adult patients (mean age 54.9 ± 14.1; male n = 93, 50.8%; body mass index (BMI) 25 ± 5.1 kg/m^2^) and aimed at investigating the food preferences of a representative sample of HD patients in order to formulate a food product aligned with their particular likes and preferences. These patients replied to a food questionnaire about their preferences regarding food consistency and taste. With the results of this questionnaire, two food products were developed in order to be suitable for use as a dialysis snack. The second sample was made up of 60 patients (mean age 60.5 ± 15.2; male n = 32, 53%; BMI 23.6 ± 4.3 kg/m^2^) from another dialysis center, who in addition to the inclusion criteria as described above, had serum albumin < 4.0 g/dL. This group participated in a sensory analysis of the developed food products. Considering that these products contained no added sugar, diabetic patients were included, but patients with gluten and/or lactose intolerance or allergies were not. Figure 1 describes the study design, protocol and patient selection.

The study protocol was approved by the Research Ethics Committee (Pedro Ernesto University Hospital, Rio de Janeiro State University, number 2.259.878). All procedures were in compliance with the principles of the Declaration of Helsinki. Informed consent was obtained from each of the participants of this study.

### Food products

The results of the food questionnaire administered to 183 HD patients are shown in Table 1 and indicate a preference for a product of solid consistency with a salty taste. Therefore, two muffin formulations of fine herbs, one enriched with whey protein concentrate (WPC) and a further muffin with textured soy protein (TSP) were developed. Both formulations were devised with limited content of K, P and Na, and were developed at the Institute of Nutrition (Rio de Janeiro State University, Brazil). The chemical composition and nutritional composition of the muffins were analyzed at the Institute of Chemistry (Rio de Janeiro State University, Brazil).

**Table 1:** Results of the food preferences questionnaire of adult patients undergoing chronic hemodialysis (n = 183)

Variable	n; %
Food consistency	
Solid	166; 90.7
Liquid	1; 0.6
Pasty	16; 8.7
Flavor	
Salty	149; 81.4
Sweet	34; 18.6

In order to compare the protein-enriched muffins with a standard muffin and investigate whether the protein-enriched muffin would show different acceptance from the standard formulation, a muffin formulation without protein was developed (M_Standard_). The formulations enriched with either WPC or TSP were developed by replacing 1/3 of the wheat flour with a protein base (M_WPC_ and M_TSP_). All ingredients were manually mixed, homogenized and oven baked at 220 °C for 30 min. The muffins were prepared according to the formulations shown in Table 2.

**Table 2:** Ingredients of standard muffin, muffin added with whey protein concentrate and muffin added with textured soy protein

Ingredients	M_Standard_	M_WPC_	M_TSP_
Refined wheat flour (g)	150	100	100
Whey protein concentrate (g)	–	50	–
Textured soy protein (g)	–	–	50
Baking powder (g)	5	5	5
Herbs^A^ (g)	2.5	2.5	2.5
Fresh garlic (g)	2.5	2.5	2.5
Refined salt (g)	1	1	1
Egg (g)	100	100	100
Whole milk (mL)	70	70	70
Soy oil (mL)	50	50	50

*M*_*Standard*_ muffin standard, *M*_*WPC*_ muffin with whey protein concentrate, *M*_*TSP*_ muffin with textured soy protein

^A^Herbs: Mix of dehydrated parsley, chives, tarragon, rosemary and thyme

### Chemical composition of the muffins

The moisture, ash, fat and protein contents of the muffins were determined according to the *Association of Official Analytical Chemists* (AOAC) [13], while carbohydrate was determined by difference [14]. Briefly, the moisture was oven dried at 105 °C to constant weight, while ash was determined by incineration of the samples in a muffle furnace at 550 °C to constant weight. Fat was determined using the Soxhlet method followed by evaporation to constant weight and protein was analyzed using the Kjeldahl method with a conversion factor of 5.7 (wheat), 6.25 (soy) or 6.38 (milk) [13].

Finally, P, K and Na from refined wheat, WPC and TSP were determined by inductively coupled plasma mass spectrometry according to the methodology of the Adolfo Lutz Institute [15]. All analyses were performed in triplicate for the process and sample. The mineral content of the remaining ingredients was obtained from the Brazilian Table of Food Composition (TACO) [16].

### Nutritional status

The group of patients enrolled in the sensory analysis (n = 60) had their nutritional status assessed by body weight and height, SGA-7p and serum albumin. The SGA-7p, translated and validated to Portuguese, was used [17], and patients were classified as well-nourished (score 7–6), mild to moderate malnutrition (score 5–3) and severe malnutrition (score 2–1). The same trained dietitian performed both the anthropometric measurements and the SGA-7p.

### Laboratory analysis

Blood samples for laboratory tests were taken in the midweek dialysis session. Serum urea (pre and post dialysis for Kt/V calculation), creatinine, albumin, potassium and phosphorus were evaluated for patients who participated in the sensory analysis (n = 60). Serum albumin was analyzed by the bromocresol green method.

### Sensory analysis

Acceptance of the muffins was assessed by applying an affective test, using the 9-point structured hedonic scale which ranged from "extremely liked" (score 9) to "extremely disliked" (score 1). Appearance, texture, taste and overall acceptance were the sensory attributes evaluated. The patients received one sample (10 g) of each muffin (WPC, TSP and Standard) coded with three random digits in order to prevent identification. Sensory analysis of the three products occurred separately and, between each of the tastings, a glass of water (50 ml) was offered to minimize the residual taste of the previous sample.

The results are expressed as the mean score for each attribute and the acceptance index (AI) was calculated as follows: AI (%) = A*100/B, where A was the mean score obtained for each formulation and B was the maximum score obtained [15]. The muffin formulations with a higher than 70% AI were well accepted [18]. The intention of consumption was also performed using a 5-point scale ranging from "I would certainly eat it" (score 5) to "I would never eat it" (score 1).

### Statistical analysis

Data are described as mean ± standard deviation, or as median and interquartile range, as appropriate, depending on the variable distribution. Categorical variables are shown as absolute values and respective percentage. Comparison of the chemical and sensory composition between the standard muffin and the protein-enriched muffins was performed by analysis of variance (ANOVA) for independent samples, with post-hoc Bonferroni or the Kruskal–Wallis test, depending on the distribution of the variable. The chi-square test was used to compare categorical variables. The statistical tests were performed using the Statistical Package for the Social Sciences (SPSS)—version 23 (Inc., Chicago, IL, USA) and a P value < 0.05 was considered statistically significant.

## Results

**Fig. 2:**
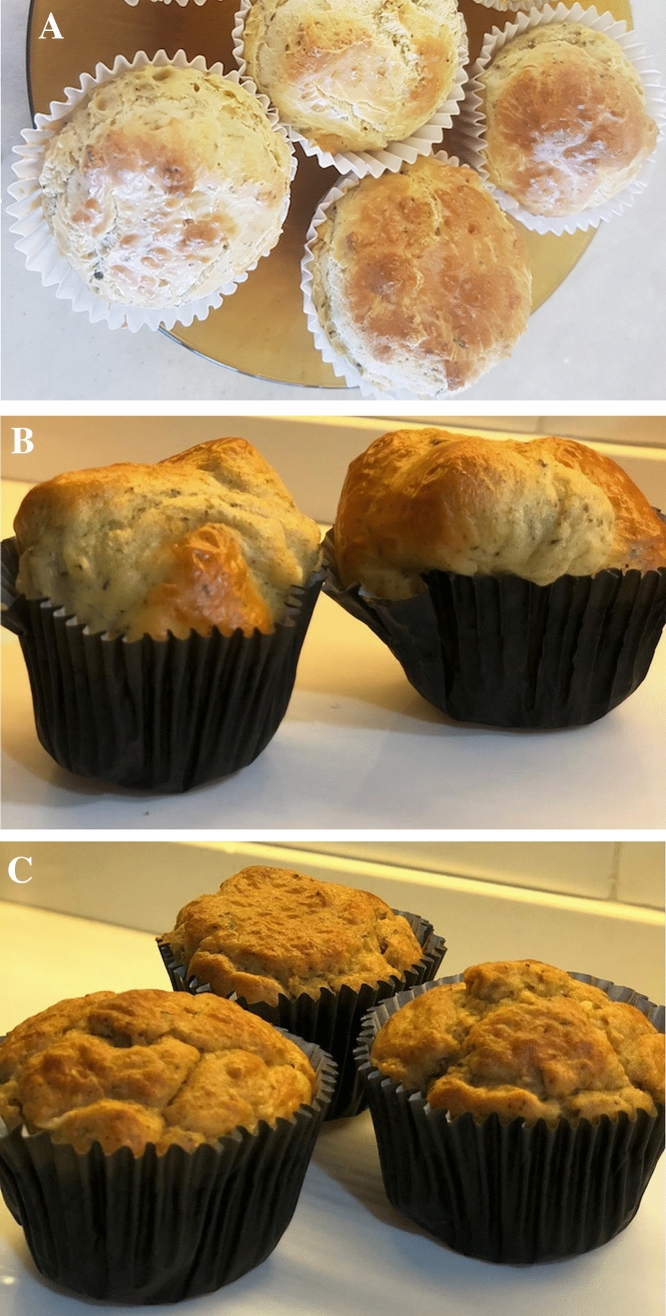
Muffins of fine herbs developed for patients undergoing chronic hemodialysis and used in the sensory analysis. **a** Standard Muffins (not enriched with protein). **b** Muffins enriched with whey protein concentrate. **c** Muffins enriched with textured soy protein

The muffins are shown in Fig. 2 and their chemical composition is listed in Table 3. No differences were observed in the moisture, ash, fat content or energy values among the 3 muffins. As expected, the protein content of the protein-enriched muffins (M_WPC_ and M_TSP_) was significantly higher than that of the M_Standard_, with the M_WPC_ showing the highest protein content. Moreover, the carbohydrate content of the protein-enriched muffins (M_WPC_ and M_TSP_) was significantly lower than that of M_Standard_. The mineral content proved adequate for HD patients, according to the CKD nutrition guidelines [19, 20]. The K, P and Na content per serving of the M_Standard_ were 83.5 mg, 48.2 mg and 183.2 mg, respectively; for the M_WPC_ these values were 107.9 mg, 48.9 mg and 183.4, respectively; and for the M_TSP_ they were 225.2 mg, 49.6 mg and 183.2 mg respectively. The estimated cost of one serving of the enriched protein muffins was USD $0.50 cents (estimated in August of 2018).

**Table 3:** Chemical composition of the muffins

Composition	Muffins (one serving = 60 g)^A^			
	M_Standard_ Mean ± SD	M_WPC_ Mean ± SD	M_TSP_ Mean ± SD	One-way ANOVA (*P* value)
Moisture (g)	19.9 ± 1.0	20.9 ± 1.1	20.5 ± 0.1	0.41
Ash (g)	1.7 ± 0.2	1.8 ± 0.2	2.1 ± 0.2	0.08
Energy (Kcal)	213 ± 2.9	208 ± 11.8	216 ± 2.7	0.41
Protein (g)	5.9 ± 0.3^a^	14.5 0.9^b^	10.8 ± 0.7^c^	< 0.001
Fat (g)	15.3 ± 1.2	14.2 ± 1.2	16.6 ± 0.4	0.08
Carbohydrate (g)	13.1 ± 2.2^a^	5.6 ± 0.8^b^	6.0 ± 1.2^b^	0.002
Potassium (mg)^A^	83.5	107.9	225.2	NA
Phosphorus (mg)^B^	48.2	48.9	49.6	NA
Sodium (mg)^B^	183.2	183.4	183.2	NA

*M*_*Standard*_ muffin standard, *M*_*WPC*_ muffin with whey protein concentrate, *M*_*TSP*_ muffin with textured soy protein, *NA* not applicable, *SD* standard deviation, *ANOVA* one-way analysis of variance analysis with Bonferroni post-hoc test

Different letters on the same line indicate statistical difference (p < 0.05)

^A^Analysis carried out in triplicate

^B^Value from the Brazilian Food Composition Table

Table 4 describes the demographic, clinical and nutritional characteristics of the HD patients that participated in the sensory analysis. In general, males and females were equally distributed, 60% of the patients had some degree of malnutrition according to SGA, and the mean serum albumin was below 3.8 mg/dL. In addition, mean serum K and P were within normal range, but 45% of the patients had hyperkalemia (serum K > 5 mEq/L) and 28% had hyperphosphatemia (serum P > 5 mg/dL). Regarding the sensory analysis, the results are shown in Table 5. All formulations were well-accepted with regard to the attributes of appearance, texture and taste, and the overall acceptance scored above 7, thus indicating good acceptance. No statistical difference between the formulations was observed. Moreover, the acceptability index was higher than 70% for all attributes. The results of the intention of consumption showed that 85%, 83.3% and 78.3% of the patients rated the M_WPC_, M_Standard_ and M_TSP_, respectively, as “I would certainly eat it”.

**Table 4:** Demographic, clinical and nutritional characteristics of the hemodialysis patients that participated in the sensory analysis (n = 60)

Variable	Values^a^
Male (n; %)	32 (53)
Age (years)	60.5 ± 15.2
DM (n; %)	13 (22)
Urea Kt/V	1.7 0.43
BMI (kg/m^2^)	23.6 ± 4.3
7p-SGA	5.2 ± 1
Malnourished^b^ (n; %)	36 (60)
Serum urea (mg/dL)	113 (97–140)
Serum creatinine (mg/dL)	8.3 (6.6–11.1)
Serum albumin (g/dL)	3.6 ± 0.3
Serum potassium (mEq/L)	5.0 ± 0.8
Serum phosphate (mg/dL)	4.2 (3.8–5.1)

*DM* diabetes mellitus, *Kt/V* dialyzer urea clearance, *BMI* body mass index, *7p-SGA* 7 point Subjective Global Assessment

^a^Values described as mean and standard deviation or as median and interquartile interval or as absolute values and respective percentage

^b^Malnourished: diagnosed by SGA-7p ≤ 5

**Table 5:** Sensory analysis and acceptability index of the muffins

Sensory Attributes	Accepted test (mean ± SD)				Acceptability index (%)		
	M_Standard_	M_WPC_	M_TSP_	P*	M_Control_	M_WPC_	M_TSP_
Appearance	7.2 ± 1.9	7.2 ± 1.8	7.1 ± 2.1	0.44	80	81	79
Texture	7.4 ± 1.9	7.3 ± 1.8	7.2 ± 2.1	0.35	82	80	79
Taste	7.4 ± 1.8	7.2 ± 1.8	7.0 ± 2.2	0.54	82	80	78
Overall acceptance	7.4 ± 1.9	7.2 ± 1.8	7.0 ± 2.1	0.59	82	85	78

*SD* standard deviation, *M*_*Standard*_ control muffin, *M*_*WPC*_ muffin with whey protein concentrate, *M*_*TSP*_ muffin with textured soy protein

P* ANOVA test (analysis of variance) for paired samples

## Discussion

This study aimed to develop food products with high amounts of energy and protein whilst being moderate to low in K, P and Na to meet the nutritional needs of HD patients, mainly those facing nutritional risk or those who are malnourished. In addition, in order to be suitable as a dialysis snack, we also aimed to develop food products that could further be produced as “home-made”, and at an affordable cost. After administering a food preference questionnaire, in which patients reported their preference for salty and solid food, a muffin with fine herbs was developed with two sources of protein, WPC and TSP, aimed at reaching two price ranges. The results pertaining to the chemical composition of the muffins showed that, compared to the M_Standard_, the M_WPC_ and M_TSP_ had significantly higher protein content, whilst retaining an adequate amount of K, P and Na for dialysis patients. Particularly with regard to K, the M_TSP_ yielded slightly over 200 mg/serving (225.2 mg), which is higher than that normally accepted for low K food (< 200 mg/serving) according to the Nutrition Kidney Disease Outcomes Quality Initiative guidelines (KDOQI) [20]. In the current study, 45% of the patients that participated in the sensory analysis had hyperkalemia (serum K > 5 mEq/L). Likewise, a lower prevalence of hyperphosphatemia (serum P > 5 mg/dL) was observed (28%). These findings suggest that the majority of patients would not need to restrict food sources of K and P when planning an intervention to treat malnutrition. However, if malnutrition and these mineral abnormalities coexist, restriction of food sources of K and P is to be adopted only where an excessive intake of these minerals is present. Particularly for the use of the muffins herein presented, the M_WPC_ is preferable to the M_TSP_ in case of hyperkalemia, and since the P content was low, both muffins can be recommended even if hyperphosphatemia occurs. In terms of cost, two servings of the enriched protein muffins cost about half as much, but have the energy and content close to that of an industrialized food supplement, rich in energy and protein and moderate in Na, K and P. Of further importance, the sensory analysis of both enriched protein muffins, as reported by 60 HD patients, showed that the muffins were well-accepted with regard to appearance, texture and taste, in addition to having a high overall index of acceptance, acceptability and a high percentage of intention of consumption. Our results confirm that these products are suitable, low-cost options for HD patients. Although the recommendation of this muffin should be individualized by the dietitian on the basis of nutritional status, appetite, tolerance and overall clinical condition of each patient, it is likely that for a patient with malnutrition on HD, two muffins per dialysis session would be adequate. As a way of stimulating healthier options that are adequate for dialysis snacks, patients who participated in the current study received a folder with the recipe and nutritional content of the muffins. Therefore, initiatives like the one presented here, that motivate patients to find “ultra-processed and additive-free” options, with recipes that can be home-made, and with educational activities that develop cooking skills, such as preparing these muffins, are important in order to motivate a healthier dietary quality and more independence in the dietary treatment. Such initiatives are aligned with those recommended by the food guide of the Brazilian population [21] and by the Italian Society of Nephrology, which include healthy lifestyle and diet [22]. This becomes more relevant when one considers that HD is a chronic and long-term treatment, and that patient involvement in his/her own treatment is key for a better outcome.

As has been well documented worldwide, the prevalence of malnutrition in HD patients, assessed by SGA-7p or MIS, ranges from 28 to 54% [1]. In the present study, 60% of patients had some degree of malnutrition as assessed by SGA-7p, a prevalence higher than that reported above, justified by the inclusion criteria of the current study—serum albumin < 4 g/dL, to select patients at nutritional risk or with malnutrition. Therefore, the patients included in the sensory analysis would show more benefit from food initiatives with increased energy and protein amounts. As is well-known and has been demonstrated, malnutrition is highly associated with poor quality of life [23, 24], increased hospitalization rates [24, 25] and higher all-case and cardiovascular mortality [24–30] Since increasing energy and protein intake is a recognized approach to treating malnutrition, strategies as the one from the current study are relevant for the clinical practice of dietitians working with end-stage kidney disease (ESKD) patients, especially because industrialized supplements are not always feasible for all patients in dialysis centers. This result shows the importance of developing food products that can increase the options of intradialytic supplementation and that match with the patient’s food preferences. Therefore, our findings regarding the preference for salty and solid snacks, which is the opposite of the characteristics of industrialized supplements that tend to be sweet and of liquid consistency, are of interest in order to explore food products with a higher chance of acceptability and adherence for long term use. This led us to develop a bakery product that has the advantage of being well-accepted worldwide, except for those patients with gluten intolerance. Moreover, in order to avoid flavor monotony; varying the types of herbs and spices, such as oregano, rosemary, dill, tarragon, cumin, garlic, paprika and onion, among others, can be used without compromising the energy, protein, Na, K and P content of the muffins. Other studies that developed non-industrialized food products for HD, although scarce, showed promising results in improving the nutritional status of patients that were malnourished [31–34].

Roy et al. [31] developed a cookie that contained ingredients from the local culture (India), containing soy protein isolate as the protein source. Compared to the standard cookie, the cookie developed in the study contained approximately 25% more fat than the standard, and 9 g of protein/100 g [31]. After an intervention with three cookies per day for 3 months, which provided 590 kcal and 10.4 g of protein/day, there was a significant increase in the anthropometric measurements (dry body weight, arm circumference, mid-arm muscle circumference, waist and hip circumference), laboratory measurements (hemoglobin, pre-dialysis urea, albumin, iron binding capacity), dietary intake [energy, protein, K, calcium (Ca), P], with a reduction in C-reactive protein (CRP), and MIS, the latter pointing to an improvement in the overall nutritional status [31]. Although this study lacked a control group, this intervention showed a positive impact by improving the nutritional status of the participants [31]. Subsequently, Caetano et al. [32] evaluated the effect of intradialytic meals during the dialysis session for 6 months in HD patients with albumin ≤ 3.8 g/dL and compared them with a control group of HD patients with normal albumin concentration. The individual meal included a protein drink with high biological value, plus one egg sandwich, providing in total 420.3 kcal and 31 g of protein/dialysis session. After 6 months, the group receiving the protein meal showed a significant increase in fat mass as compared to the control group [32]. In another study with a randomized controlled design, conducted in HD patients, protein-enriched meals (200 mL of milk and two egg whites), provided during the dialysis session for 3 months and yielding a total of 147.6 kcal and 13.7 g of protein/dialysis session, indicated that the group receiving the protein-enriched meal showed significant improvement in serum albumin levels when compared to the control group [33]. Finally, in a recently published pilot study, Choi et al. [34] provided meals containing approximately 30 g protein and 1/3 of the recommended daily Na, K, P and liquid to HD patients during 25 consecutive dialysis sessions. They showed that, although no change in nutritional status was observed, the group receiving the meal did not show an increase in symptomatic hypotension events associated with the meals. This finding suggests that there were no clinical complications due to eating during the dialysis session [34]. Altogether, these results are supportive of the idea that non-industrialized food products are both plausible and a more likely and affordable strategy towards treating malnutrition in HD patients.

Although our study suffers from the limitation of not planning for an intervention to examine whether the muffins of fine herbs developed an efficient option for treating malnutrition, some important strengths can be listed: Firstly, we investigated, among a group of HD patients, which characteristics they would like to have in a food product in order to align it with the patient’s preference and expectations; secondly, we evaluated the safety of these products by assessing their chemical composition; thirdly, the enriched protein muffins were made with affordable ingredients to allow their use among most patients and over a long period of time. Finally, we performed a detailed sensory analysis of the muffins to investigate whether these products were well-accepted by the patients. When all factors are taken together, we believe that our findings can be used to support other clinical practitioners towards developing food products, in accordance with their local possibilities, and even to test the use of the current food products in other dialysis clinics.

In conclusion, the current study presents the feasible and affordable option of two muffins of fine herbs to function as snacks for dialysis patients. These products were shown to be appropriate for HD patients, with adequate amounts of energy, protein, K, P and Na. In addition, the muffins had a high degree of acceptance, as assessed by the sensory analysis. The effectiveness of these products to treat malnutrition remains to be further investigated in a subsequent study.

## Data Availability

The data generated and analyzed during the present study are under the domain of the corresponding author and will be made available upon request and evaluation.
